# Small-Angle X-Ray and Neutron Scattering on Photosynthetic Membranes

**DOI:** 10.3389/fchem.2021.631370

**Published:** 2021-04-19

**Authors:** Dainius Jakubauskas, Kell Mortensen, Poul Erik Jensen, Jacob J. K. Kirkensgaard

**Affiliations:** ^1^ X-ray and Neutron Science, Niels Bohr Institute, University of Copenhagen, Copenhagen, Denmark; ^2^ Department of Food Science, University of Copenhagen, Copenhagen, Denmark

**Keywords:** SAXS, SANS, thylakoids, photosynthesis, structural modeling, small-angle scattering

## Abstract

Ultrastructural membrane arrangements in living cells and their dynamic remodeling in response to environmental changes remain an area of active research but are also subject to large uncertainty. The use of noninvasive methods such as X-ray and neutron scattering provides an attractive complimentary source of information to direct imaging because *in vivo* systems can be probed in near-natural conditions. However, without solid underlying structural modeling to properly interpret the indirect information extracted, scattering provides at best qualitative information and at worst direct misinterpretations. Here we review the current state of small-angle scattering applied to photosynthetic membrane systems with particular focus on data interpretation and modeling.

## 1 Introduction

**FIGURE 1:**
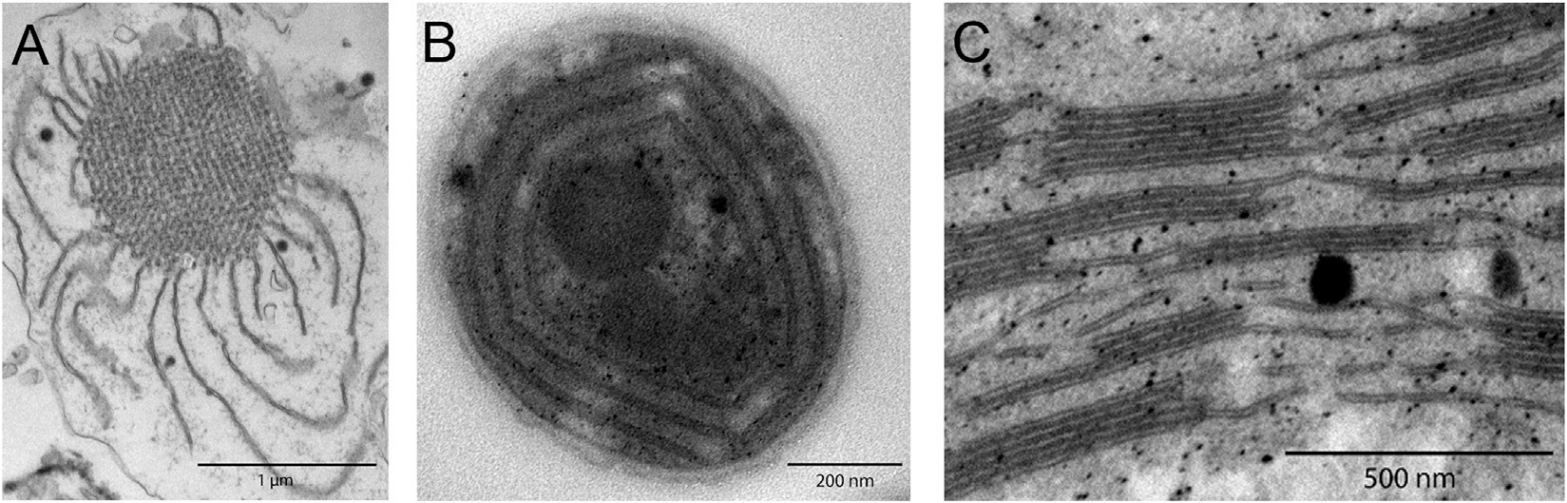
TEM images of photosynthetic membrane systems, revealing complex structural characteristics. **(A)** Etiolated maize prolamellar body. **(B)** Concentric *Synechocystis* sp. PCC 6803 cyanobacterial thylakoids. **(C)** Higher plant grana stacks of *Arabidopsis* Col0, interconnected by stromal thylakoids.

The lamellar nature of thylakoids was shown in the pioneering works of Menke in 1940 (Menke, 1940a; Menke, 1940b) who studied the inner structure of chloroplasts by light and electron microscopies. From these studies, it became evident that photosynthetic membranes are not randomly dispersed within the cell but organize into highly complex ultrastructures. Figure 1 depicts representative transmission electron microscopy (TEM) images of photosynthetic membrane systems: from the remarkably intricate network structure of the prolamellar body found inside developing etioplasts to individual cyanobacterial thylakoids and finally stacked grana thylakoid arrays in higher plants. The structural regularity in these TEM images suggests that structural information can be extracted from X-ray or neutron scattering methods. In this review, we focus on small-angle scattering techniques: small-angle X-ray (SAXS) and neutron (SANS) scattering.

Small-angle scattering enables investigating structures of ca. 1–200 nm in near *in vivo* conditions and is widely applied in structural biology and soft matter sciences. Small-angle scattering complements various microscopic techniques, such as TEM (Menke, 1960; Paolillo and Paolillo, 1970; Staehelin, 1986; Austin and Staehelin, 2011; Armbruster et al., 2013; Heinz et al., 2016; Kowalewska et al., 2016; Wood et al., 2018; Kowalewska et al., 2019; Wood et al., 2019; Li et al., 2020), scanning electron microscopy (Mustárdy and Jánossy, 1979; Armbruster et al., 2013), cryo-EM (Ford et al., 2002; Kirchhoff et al., 2011; Engel et al., 2015), cryoelectron tomography (Shimoni et al., 2005; Austin and Staehelin, 2011; Daum and Kühlbrandt, 2011; Kouřil et al., 2011; Ford and Holzenburg, 2014; Bussi et al., 2019; Rast et al., 2019), atomic force microscopy (Kaftan et al., 2002; Sturgis et al., 2009; Sznee et al., 2011; Grzyb et al., 2013; Onoa et al., 2014), confocal laser scanning microscopy (Kowalewska et al., 2016; Mazur et al., 2019), and live cell imaging (Iwai et al., 2014; Iwai et al., 2016). The first scattering studies on photosynthetic membranes were performed in 1953 by Finean et al. (1953) and has continued ever since. There are currently about 40–50 works published on scattering from photosynthetic systems—photosynthetic bacteria, diatoms and other algae and of course from higher plants. Small-angle scattering has been used to investigate structure and dynamic changes of thylakoid membrane systems of plants (Finean et al., 1953; Kratky et al., 1959; Kreutz and Menke, 1960a; Kreutz and Menke, 1960b; Kreutz and Menke, 1962; Kreutz, 1963a; Kreutz, 1963b; Kreutz, 1964; Sadler, 1976; Li, 1979; Sadler and Worcester, 1982a; Sadler and Worcester, 1982b; Diederichs et al., 1985; Garab et al., 1997; Kirkensgaard et al., 2009; Nagy, 2011; Nagy et al., 2011;Nagy et al., 2013; Ünnep et al., 2014b; Herdean et al., 2016; Ünnep et al., 2017; Zsiros et al., 2020; Ünnep et al., 2020), protists (Sadler et al., 1973; Worcester, 1976; Sadler and Worcester, 1982a), diatoms (Nagy et al., 2011; Nagy et al., 2012), photosynthetic bacteria (Pape et al., 1974; Hodapp and Kreutz, 1980; Liberton et al., 2013b; Ünnep et al., 2014a; Li et al., 2016; Stingaciu et al., 2016; Eyal et al., 2017; Jakubauskas et al., 2019), algae (Nagy et al., 2011; Nagy et al., 2012; Nagy et al., 2014), light harvesting complexes (Smidijiev et al., 2000; Tang and Blankenship, 2012), phycobiliproteins (Golub et al., 2017), and higher-plant prolamellar bodies (Williams et al., 1998; Selstam et al., 2007). The aim of applying small-angle scattering in biological sciences is to investigate structural changes of the biological system and correlate them with underlying physiological processes *in vivo*. This article critically reviews the current state of small angle scattering applied to photosynthetic membrane systems with a three-fold agenda: (1) to describe the basics of the method and present an overview of existing small-angle scattering results on photosynthetic membrane systems, (2) to discuss scattering results and their correlation with microscopy, critical points of result interpretation, and method limitations, and (3) to envision the development of the small angle scattering method with focus on data analysis and modeling in the field of photosynthetic membrane systems. The review is organized as follows: small-angle scattering terminology and relevant background is introduced in Section 2. Scattering results from plant, cyanobacterial, algae, and diatom thylakoid membranes including dynamic changes during illumination are critically evaluated in Sections 3–6. Finally, an outlook is presented in Section 7.

## 2 Small-Angle X-Ray and Neutron Scattering

### 2.1 The Small-Angle Scattering Experiment

**FIGURE 2:**
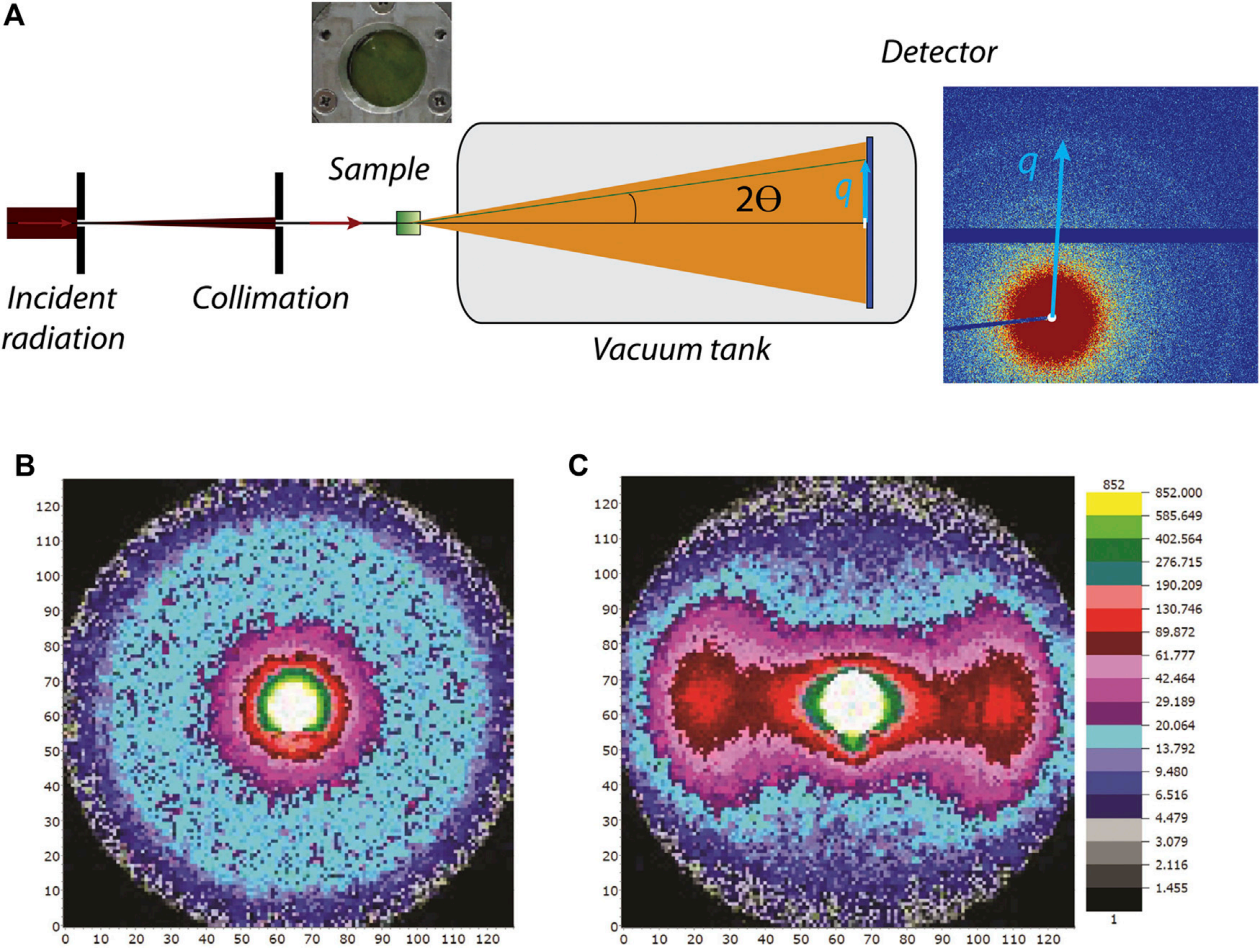
**(A)** General experimental setup. Incident radiation is collimated and penetrates the sample (green box). Scattering arising in 2*θ* direction and the resulting scattering vector *q* are depicted in light blue. A beamstop (white) blocks the primary radiation. **(B)** Isotropic 2-dimensional scattering pattern from nonaligned system. **(C)** Nonisotropic 2-dimensional scattering pattern from a magnetically aligned system.

**FIGURE 3:**
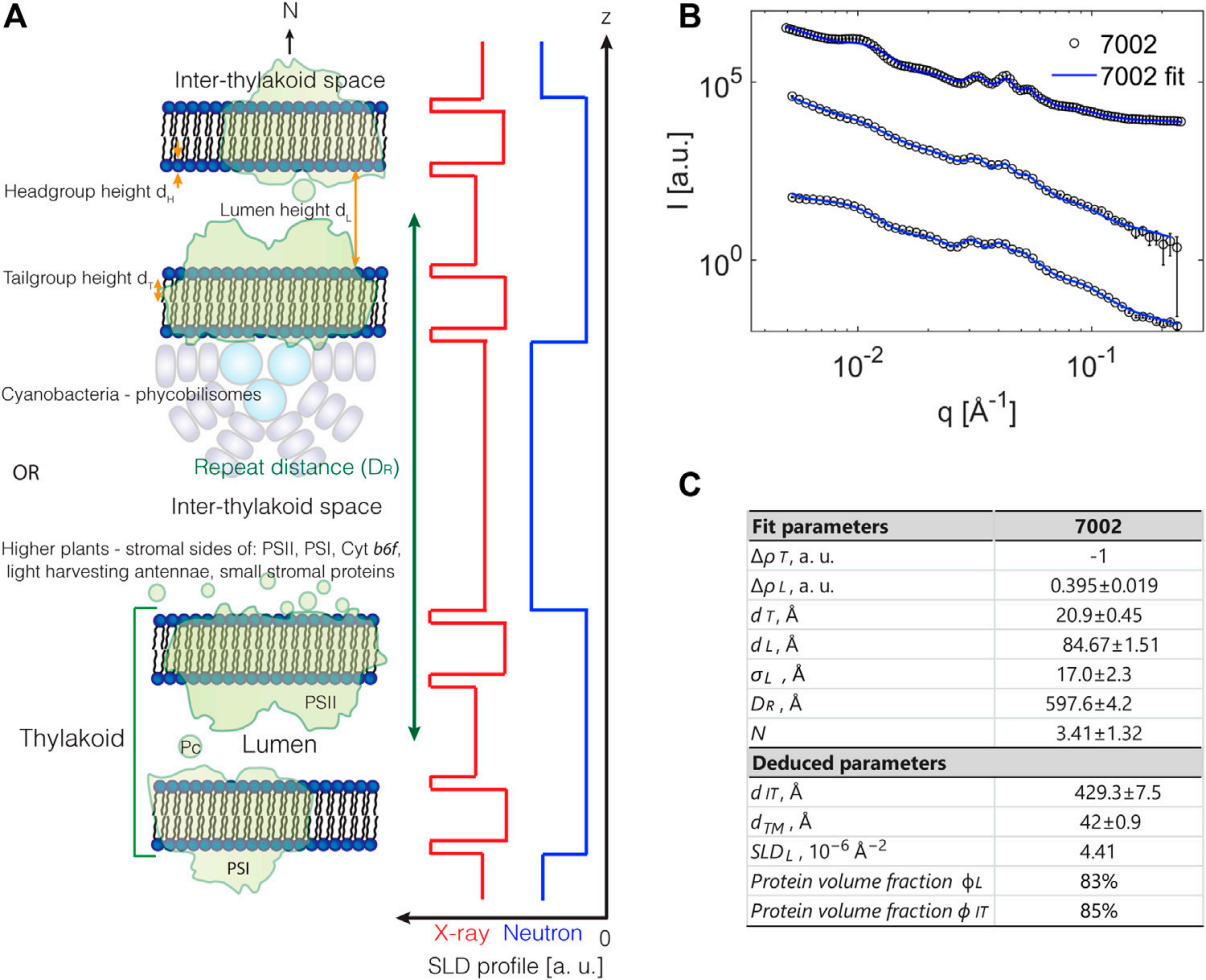
**(A)** Illustration of structural model for a photosynthetic membrane system with a stack of double layered thylakoids. Neutron (light blue) and X-ray (red) SLD profiles are schematically depicted. **(B)** Full *q*-range model fits to SANS data from three independent replicas of *Synechococcus* sp. PCC 7002 [from Jakubauskas et al. (2019)]. **(C)** Extracted structural numbers from fits in B.

The concepts described below are valid for both X-rays and neutrons; however, certain differences arise due to the different physical nature and interactions of photons and neutrons with materials (see Table 1). In a material, either the electrons (for X-ray scattering) or nuclei (for neutron scattering) interact with the incoming radiation and deflect X-ray photons or neutrons from their original path. The electrons or nuclei become sources of elastically scattered secondary waves, whose intensities are registered by a detector as a function of the angle relative to the incoming beam (see Figure 2A) (Glatter and Kratky, 1982; Willis and Carlile, 2009). Contemporary detection systems typically produce a 2-dimensional output as shown in Figure 2. If a sample scatters azimuthally isotropic (Figure 2A), the 2-dimensional pattern is centrosymmetric (Figure 2B). For analysis, these 2-D patterns are typically azimuthally averaged and collapsed into a 1-dimensional curve (Figure 3B) showing scattering intensity as a function of the scattering vector magnitude:

$$q=\left|q\right|=\frac{4\pi }{\lambda }\sin\theta ,$$ (1)

where λ is the wavelength of the incident radiation (for neutrons the de Broglie wavelength) and θ is half the scattering angle (see Figure 2). Thus, the scattering vector magnitude is a direct measure of the scattering angle, but normalized by the wavelength of the radiation. These 1D scattering data are analyzed analytically or numerically to reveal the underlying structure, as described below. For nonisotropic systems (Figure 2C), sector averaging can be employed to yield 1-dimensional curves for each of the sectors, but in some cases analysis is performed directly on the 2-dimensional dataset. By combining Eq. 1 with Bragg’s law for periodic structures:

$$n\lambda =2D_{R}\sin\theta \;\left(n=1,2,3,\ldots \right),$$ (2)

a direct relation between *q* and a real-space structural periodicity $D_{R}$ is obtained:

$$D_{R}=\;\frac{2\pi n}{q}.$$ (3)

**TABLE 1:** Differences and practicalities of SAXS and SANS experimental techniques [adapted from Tang and Blankenship (2012)].

	SAXS	SANS
Interacting field	Electrons	Nuclei
Incident beam wavelength, Å	0.8–1.6	2.0–25.0
Flux of the source (particles/s/mm^2^)	Medium to high (10^8−9^–10^11^)	Very low to low (10^5^–10^8−9^)
Coherent scattering length density, 10^–12^ cm	H: 0.28, D: 0.28	H: −0.374, D: 0.667
Sample volumes required in 1–2 mm path length cell	20–30 μL	150–500 μL
Radiation damage to the sample	Low for laboratory sources, high for synchrotron sources	Low
Structural information extracted for individual	No (electron density average of the entire sample)	Yes (lipid, nucleic acid, protein
Moieties in multicomponent systems		can be investigated separately)
Contrast variation use	Rare	Common
Resolution	Low-medium	Low-medium
Experimental facilities	Laboratory and synchrotron radiation sources	Large facilities only

The fundamental inverse relation between angles and distances is defined in Eqs 1 and 3: larger angles (larger *q*) probe smaller distances and vice versa. The integer *n* from Bragg’s law appearing in Eq. 3 is called the “peak order” and indicates that a certain distance $D_{R}$, repeating in the material, gives rise to a series of peaks in the reciprocal space, ideally one for each value of n=1,2,3,…. For an ordered lamellar stack, all peaks are placed equidistantly. For more complex structures, different peak positions reflect other crystallographic symmetries and require more detailed analysis. Once a scattering intensity pattern is recorded and corrected for background contributions, structural sample parameters can be extracted from the scattering pattern by means of modeling as described below.

### 2.2 Scattering Length Density and Contrast

**FIGURE 4:**
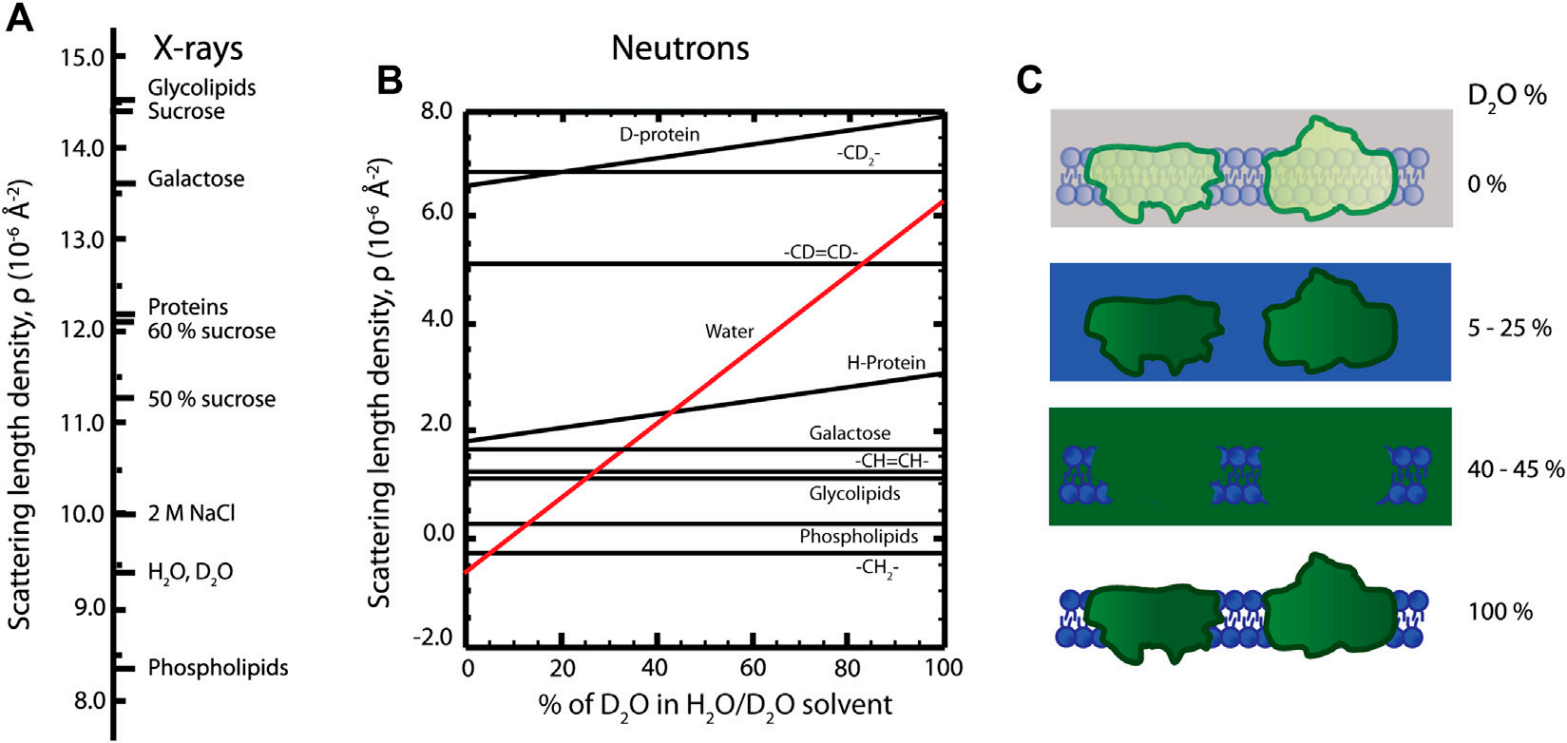
**(A)** X-ray scattering length densities for various molecules. **(B)** Dependence of neutron scattering length densities of lipid and protein moieties on D_2_O amount in the sample. **(C)** Contrast variation technique, where a photosynthetic membrane is visualized in different D_2_O buffers. The signal from lipid (5–25% D_2_O) or protein components (40–45% D_2_O) is “masked out.” The total scattering signal is enhanced in 100% D_2_O, as indicated by more intense colors than in 0% D_2_O.

In analogy to scattering of visible light where differences in refractive index give rise to scattering, for example air-material contrast, differences in *scattering length density* enable one to “see” material constituents with X-rays and neutrons. The scattering length density describes the degree of interaction between the sample molecules and the incoming radiation and thereby quantifies the scattering power of different molecular components (see Figures 4A,B). In terms of scattering intensity, the relevant concept becomes the *contrast*—the difference in scattering length density between different components. For X-rays, contrast arises from variations in the electron density of the material, and for neutrons the contrast arises from the different atomic nuclear structures.

The different physical nature of the two methods is advantageous and complementary in ultrastructural analysis—SAXS and SANS enable extracting different structural information from the same sample. The X-ray scattering power of atoms increases roughly linearly with atomic number while for neutrons the variation is less systematic and differs significantly between different isotopes (Sears, 1992). This isotope difference is particularly important for biological and soft matter samples, since the exchange of hydrogen (H) atoms with the heavy-hydrogen isotope deuterium (D) allows fine-tuning the neutron scattering contrast, hence called contrast variation. For example, if the scattering length density of a surrounding medium is equal to the scattering length density of a specific molecular component, no scattering is observed from that component—we say that the scattering has been “matched out.” In its simplest form, mixing H_2_O- and D_2_O-based buffers in specific ratios enables enhancing or diminishing the contrast of different cellular components (Serdyuk et al., 2007; Heller, 2010) (Figure 4B). To exemplify, the contrast variation technique allows an individual investigation of either lipid or protein components in a complex biological membrane system. In order to match out membrane lipids, 5–25% D_2_O containing solutions are used, and 40–45% D_2_O containing solutions are used to match out protein components of the membrane (Figure 4C). So by designing a series of measurements with varying contrasts, one can effectively build up a series of structural snapshots which allows to extract detailed information very hard to obtain in other ways. Part of such an experimental design is to estimate the scattering length densities of the sample material; however, calculating precise scattering length densities for biological systems is not straightforward, since the exact protein and lipid composition of the membrane, their volume fractions, protein H-D exchange degree, membrane-associated water content, and solvent composition need to be known. Some recently calculated scattering length density values of thylakoid membranes are given in Table 2 (Jakubauskas, 2016; Jakubauskas et al., 2019).

**TABLE 2:** Scattering length densities of thylakoid components [taken from Jakubauskas (2016) and Jakubauskas et al. (2019)].

	Neutron SLD, Å^−2^	X-Ray SLD, Å^−2^
Lipid headgroups: plants	1.77 × 10^–6^	1.30 × 10^–5^
Lipid tailgroups: plants	1.36 × 10^–6^	1.12 × 10^–5^
Lipid headgroups: cyanobacteria	1.83 × 10^–6^	1.19–1.34 × 10^–5^
Lipid tailgroups: cyanobacteria	1.33 × 10^–6^	1.12 × 10^–5^
Thylakoid proteins (10% H-D exchange): plants	2.43 × 10^–6^	1.22 × 10^–5^
Thylakoid membrane: cyanobacteria	1.58 × 10^–6^	1.23 × 10^–5^
Lumen: cyanobacteria	4.29–4.41 × 10^–6^	1.15–1.18 × 10^–5^
Interthylakoid space: cyanobacteria	3.61–4.43 × 10^–6^	1.16–1.19 × 10^–5^
Chloroplast average	5.35 × 10^–6^	9.98 × 10^–5^
Stroma average	6.34 × 10^–6^	9.44 × 10^–5^
D_2_O	6.393 × 10^–6^	9.455 × 10^–5^
H_2_O	−5.61 × 10^–6^	9.469 × 10^–5^

### 2.3 Small Angle Scattering Analysis

Typical interpretations of small angle scattering data on photosynthetic membranes have so far been limited to the estimation of the average thylakoid membrane repeat distance from the observed peak position by directly applying Eq. 3. This method provides an approximation of the average spacing between thylakoid membranes and is typically used to follow system behavior in changing conditions: for example, illumination intensity, temperature, pH, or different ionic strength (see below). However, a number of factors are not accounted for in such an approach: interthylakoid space/lumen or membrane bilayer thicknesses, the number of thylakoid layers, the degree of system orientation, and, for neutron scattering in particular, instrument resolution effects. Therefore, more sophisticated analyses of scattering data on photosynthetic systems are required. Theoretical calculations of Kirkensgaard et al. (2009), based on simulated scattering patterns, suggested a possible route for further modeling which was recently demonstrated to provide a framework for the analysis of the full scattering curve from cyanobacterial membranes (Jakubauskas et al., 2019). The general equation for the scattering intensity of particles in solution is

$$I\left(q\right)=\Delta \rho^{2}\Phi_{p}V_{p}P\left(q\right)S\left(q\right).$$ (4)

Here $\text{Δ}\rho^{2}$ is the contrast of the particles relative to the solution, $\Phi_{p}$ the volume fraction of the particles in the solvent, and $V_{p}$ the particle volume. The terms P(q) and S(q) are named the *form factor* and *structure factor*, respectively. The form factor describes the scattering from an individual particle or unit cell, and the structure factor describes the interactions between these particles/units. The modeling of small-angle scattering data requires either an educated choice of the precise expression to use for the form and structure factors or, in cases where such expressions does not exist, the derivation of them which can be a complicated matter (Oliveira et al., 2012). The advantage of a full-scale modeling is a much more detailed idea about the structural organization of the system. Compared to simply applying Eq. 3, which provides the overall stacking repeat distance, the data analysis can now also give information on the internal distribution of distances as described below and take into account instrument effects and polydispersity for example. The overall goal of modeling thylakoid scattering is to construct a mathematical model that incorporates these parameters and properly reproduces the experimentally measured scattering curve without undue overparametrization. As described in detail in Jakubauskas et al. (2019), such a model is possible to construct and captures the main features of scattering from stacked thylakoids over basically the full *q*-range obtained in a SAXS/SANS experiment.

### 2.4 A Full Scattering Model

In Figure 3A a structural model for a stacked thylakoid system is illustrated. A complication in photosynthetic membrane systems is that the repeating unit is usually not a single bilayer sheet, but a double bilayer separated by inner and outer liquid compartments, the lumen and the interthylakoid space, respectively. Thus, the form factor needs to reflect this organization while the structure factor should account for the stacking order of these units. In principle, the form and structure factor are coupled, but for highly anisotropic systems like the membrane stacks described here they can be treated separately (Oliveira et al., 2012) as also demonstrated by simulations in Jakubauskas et al. (2019). The stacking order of lamellar systems is known to be well described by the structure factor from Nallet et al. (1993) while the double bilayer form factor is derived explicitly in Jakubauskas et al. (2019). As shown in Figure 3A, the form factor is built from a step model, or “box” model, which is probably the simplest possible description of the scattering density variation. Other options include smoother Gaussian variations and multilayer models (Oliveira et al., 2012). Regardless, the model presented in Figure 3A can be considered a reasonable first approach of modeling this complex biological system, and as shown in Figure 3B, the model fits the data very well. The parameters entering the form factor equations are all the “local” distances of the double bilayer structural unit cell, that is, the headgroup, tailgroup, and lumen heights along with the scattering length densities of those domains. The structure factor on the other hand relies on the “global” parameters: the number of layers in the stack, the overall stacking repeat distance, and finally a measure of the membrane rigidity. The final intensity is also influenced by the instrument resolution, inherent sample disorder, and dispersity in the number of layers of each stacked system, dispersity in the water layers, etc., all of which affect the “clarity” of scattering peaks and have to be accounted for. The output of the fitting routine based on the structural model shown in Figure 3B is summarized in Figure 3C, showing that the overall stacking repeat distance is around 60 nm, the average number of layers in a stack ca. 4, the lumen width ca. 8–9 nm, the membrane thickness 4–5 nm, and the interthylakoid space size ca. 45 nm. Finally, from the relative scattering length density levels of the inner and outer liquid compartments, it is concluded that the lumenal protein content is higher than that in the thylakoid membrane, but lower than that of the interthylakoid space.

Despite the reasonable fits to the data provided by the model presented in Jakubauskas et al. (2019), this is not a universal answer to scattering analysis from photosynthetic membrane systems, but it provides one solution. As already hinted above, one could make other choices for the precise implementation of the structure and form factors or the implementation of polydispersity could be done differently. Finally, there are also inherent assumptions which may turn out to be nonoptimal; one example is the form factor where the individual bilayers in the above modeling are assumed to be symmetric which is probably biologically unrealistic. The combination of next-generation neutron facilities and optimized sample preparations could mean that such increased details will become possible to model and extract in the future.

### 2.5 Scattering versus Microscopy

Structural studies of photosynthetic membranes are dominated by microscopy, so we briefly comment on the differences between such direct imaging methods and the indirect methods provided by scattering. Electron microscopy allows investigating an ensemble of individual sample features in Å resolution, but the statistical analysis of ultrastructures from TEM micrographs requires choosing a number of well-preserved and representative structures from the sample volume of the order of approximately 5 × 10^–7^ mm^3^. To compare, a relatively large sample volume of 0.1–1 mm^3^ is probed by X-rays or neutrons simultaneously and a statistical low-resolution structure of the total-volume averaged system is obtained. Microscopy methods are therefore complementary to scattering in accounting for sample heterogeneity, as minute differences of individual structure are clearly observed. In electron microscopy studies, artifacts from sample preparation are common due to fixation, dehydration, and image contrast (Table 3). This is contrary to scattering methods where the sample is either in a natural state, measuring directly on a leave for example, or in controlled conditions closely mimicking or perturbing the natural conditions. Besides requiring a minimal sample preparation, scattering methods enable a relatively quick system analysis under near-native conditions and importantly allows following sample dynamics. The investigation of sample dynamics on the nanometer length scale *in situ* is only possible using scattering methods, as electron microscopy requires sample immobilization, and light super-resolution microscopy techniques do not yet provide sufficient resolution (Iwai et al., 2014; Iwai et al., 2016). Cryomicroscopy techniques allow preserving an *in vivo*-like environment, but the investigation of thick samples (e.g*.*, the entire grana stack (Kouřil et al., 2011) is yet impossible due to method restrictions and the generally insufficient contrast of membranes. Although being straight forward to execute in principle, scattering methods also have challenges. Primarily, obtaining sharp Bragg peaks on photosynthetic membranes in physiological conditions (large protein content, required H-D contrast adjustments, measurements in room temperature, and high osmolarity) can be demanding. Large system inhomogeneity smears peaks and burdens precise structural parameter calculations. Centrifugation, controlled drying (Kratky et al., 1959; Kreutz and Menke, 1960b; Diederichs et al., 1985), or application of an external magnetic field (Geacintov et al., 1972; Sadler, 1976; Nagy et al., 2011; Posselt et al., 2012; Nagy et al., 2013) can be used to increase internal order of the sample during the scattering experiment but might complicate modeling where there typically is an assumption of isotropy and of course also might alter the natural state of the system. Overall, small-angle scattering and microscopy methods complement each other, and their parallel usage is advocated. Complementary investigation of the same sample with both techniques provides detailed structural information accounting for sample inhomogeneities (electron microscopy) and following sample behavior *in vivo* (scattering).

**TABLE 3:** Comparison of scattering and microscopy techniques (Schnablegger and Singh, 2013).

Feature	Scattering	Electron microscopy
Space	Reciprocal (inverse)	Real (direct)
Resolution	Averaged sample details on the nm-scale	Local details on the nm-scale
Local structure details	Cannot be extracted	Can be extracted
Results	Representative of the entire sample average	Unique, but may not represent the entire sample
	Result interpretation is ambiguous	Average structures are hard to obtain
Preparation/experimental artifacts	Scarce in *in vitro* experiments	Artifacts inherent with chemical fixation
	Sample shall not degrade/change during measurement	Artifacts scarce for cryofixation methods
		Overall cryo-EM contrast is lower than that of TEM

## 3 Cyanobacterial Thylakoids

**FIGURE 5:**
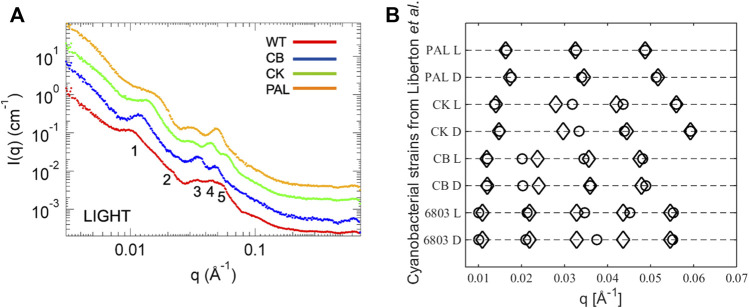
**(A)** X-ray scattering curves of cyanobacterial systems: *Synechocystis* sp. PCC 6803 (WT), CB, CK, PAL mutants. **(B)** Plot of the peak positions from Liberton et al., 2013b. Circles are data values as reported and diamonds are the *q* values calculated using the Bragg equation (Eq. 2) with the lowest *q* value as the first-order peak.

The ultrastructure of thylakoids from cyanobacteria is characterized by various arrangements of sheet-like membrane layers subject to the confinement of the surrounding cell wall. These strain-dependent arrangements are either concentric layers neighboring the cell periphery or near-radial distributions emanating from focal points on the cell membrane (Olive et al., 1997; Rast et al., 2015) (see Figure 1). From TEM data, the thylakoid repeat distance for *Synechocystis* sp. PCC 6803 (WT) and photosynthetic mutants is 340–550 Å and 430 Å for *Halomicronema hongdechloris* (Liberton et al., 2013b; Li et al., 2016). In terms of scattering studies, the wild-type strain *Synechocystis* sp. PCC 6803 is by far the most studied cyanobacteria (Liberton et al., 2013a; Liberton et al., 2013b; Ünnep et al., 2014a; Stingaciu et al., 2016). Liberton et al. (2013b) recently described neutron scattering on *Synechocystis* sp. PCC 6803 and three mutant strains with various degrees of phycobilisome deficiency (see Figure 5A). In Stingaciu et al. (2016), Stingaciu et al. (2019), the work was expanded with inelastic neutron scattering probing the dynamics of the membranes under different illumination conditions correlating the membrane mobility with photosynthetic activity. This analysis indicates a significantly softer membrane under dark conditions, supporting the result obtained for *Chlamydomonas* (Nagy et al., 2014). Finally, Li et al. (2016) recently published a study on *H. hongdechloris* where SANS work on the intact cells complements microscopy work in establishing a structural understanding of a new cyanobacterial complex showing far-red light induced decrease of thylakoid repeat distance.

Common in all these SANS studies is that the data analysis and interpretation are based on peak position readings with no underlying structural model. The interpretation leans on TEM images and treats all peaks as the first-order Bragg peaks, which we will argue in the following to be erroneous. To prove our point, in Figure 5B we plot the reported peak positions from Liberton et al. (2013b) together with peak predictions calculated from Eq. 2 with the lowest *q* value taken as the first-order peak, that is, simply treating the cyanobacterial membrane system as a stack of thylakoid lamellae. Judging from higher order peak positions, almost all data points fit with this obvious explanation. The main outliers are the second-order peaks from the CK and CB mutants, but on inspection of the original scattering curves there are no immediately apparent peaks visible, so these particular peak positions are to be regarded with a high level of uncertainty. Given the broadness of the peaks, the general noise level in the biological systems, and the neglect of form factor effects in this approach, it is hardly surprising that there are some deviations from a perfect Bragg lattice. Thus, we challenge the correlations done in Liberton et al. (2013b), where the individual peaks are interpreted to originate from specific distances in the membrane to be speculative, as all peaks clearly originate from the fundamental repeat distance of the lamellar stack which without invoking a structural model is the only information available. Nevertheless, two conclusions can be drawn from the existing cyanobacterial scattering experiments: light induces a slight shrinkage in the overall lamellar repetition which is correlated with the size of the antenna system. Knowing that plant chloroplasts are evolutionarily derived from cyanobacteria, one might expect a similar behavior of plant thylakoid membranes.

## 4 Plant Thylakoids

The first SAXS experiment on isolated osmium-fixed *Aspidistra* chloroplasts (Finean et al., 1953) indicated the existence of a structure with a repeat distance of 250 Å, and a similar $D_{R}$ was measured by Kratky et al. (1959) for *Allium porrum* chloroplast pellets. Kreutz et al. measured X-ray scattering of the thin layer of dried chloroplast pellets from *Antirrhinum majus* (Kreutz and Menke, 1960a; Kreutz and Menke, 1960b; Kreutz, 1963b). Isotropic scattering curves with peaks corresponding to the repeat distances of 177–248 Å, which putatively occurred from the ordered-layered thylakoid structure, had been obtained (Kreutz and Menke, 1962; Kreutz, 1964). A wide variation of repeat distance values from early experiments has been explained by different sample preparations and different degrees of thylakoid membrane swelling (Kreutz, 1970). Common in all early works is that scattering was used to investigate the composition and internal structure of an individual thylakoid membrane and not thylakoid membrane stacking. A systematic work trying to explain thylakoid membrane scattering was conducted by Sadler et al. (1973), Sadler (1976), on isolated chloroplasts from *Euglena gracilis* and spinach using aligned thylakoid pellets by centrifugation, partial dehydration or external magnetic field and measured X-ray or neutron diffraction: four orders of diffraction peaks having a lamellar periodicity of 165–170 Å were observed (Sadler et al., 1973). Complementing X-rays with neutron scattering experiments in a magnetic field, Sadler et al. suggested a realistic thylakoid ultrastructure model with a thylakoid repeat distance of 240–250 Å (*Euglena*) or 170–190 Å (spinach) and thylakoid membrane thickness of 50 Å (Sadler and Worcester, 1982a; Sadler and Worcester, 1982b). These values are comparable with the current (cryo-)electron microscopy measurements (Shimoni et al., 2005; Kirchhoff et al., 2011). Overall, the main outcomes of early scattering investigations were establishing the protein, lipid, and pigment arrangement in the thylakoid membrane (Kreutz, 1970; Sadler and Worcester, 1982b); estimating the thylakoid membrane thickness (Li, 1978); and providing the understanding that both the interthylakoid space and thylakoid lumen are hydrophilic compartments (Sadler and Worcester, 1982a).

### 4.1 Isolated Thylakoids

Isolation upconcentrates thylakoid membranes in the sample and reduces scattering from other plant cell components (i.e., cell wall, endoplasmic reticulum membranes, etc.), which makes the sample more pure and the interpretation of the scattering curve easier. However, an osmotic environment of an isolated sample differs from the thylakoid environment *in vivo*. Therefore, measurements on isolated and *in vivo* thylakoids are not equivalent—thylakoids swell in hypotonic (Deamer and Packer, 1967; Opanasenko et al., 1999) and shrink in hypertonic solutions (Robinson, 1985; Posselt et al., 2012; Ünnep et al., 2014b). As shown by scattering experiments (Finean et al., 1953; Kratky et al., 1959; Kreutz and Menke, 1960a; Kreutz and Menke, 1960b; Posselt et al., 2012; Ünnep et al., 2014b; Herdean et al., 2016), different sample treatments yield different thylakoid repeat distances and increased thylakoid disorder (Sadler and Worcester, 1982b) due to osmolarity and ionic force-related changes. However, due to relatively easy and fast measurements, scattering can be used to improve thylakoid or chloroplast isolation procedures with the aim of finding buffers where thylakoid membranes closely resemble the situation *in vivo* (Ünnep et al., 2014a). For example, from scattering experiments NaCl is suggested to be a better osmoticum than sorbitol for thylakoid isolation (Ünnep et al., 2014a; Ünnep et al., 2014b).

### 4.2 Plant Leaves

To avoid thylakoid ultrastructure changes due to isolation, scattering experiments on intact plant leaves have been performed. In the first SAXS experiment, *Allium porrum* (leek) leaves were stacked perpendicularly to the X-ray beam to suppress cell wall scattering (Kratky et al., 1959; Kreutz and Menke, 1962). Measuring scattering of both green and variegated snapdragon leaf parts (Wild, 1957) allowed subtracting background scattering and obtaining scattering solely from thylakoid membrane stacks (Kreutz, 1964). SANS measurements of the yellow part of *Schefflera arboricola* leaf or red bracts of *Euphorbia pulcherrima*, where thylakoid stacking is either absent or disordered, also did not exhibit scattering peaks (Ünnep et al., 2014a).

**FIGURE 6:**
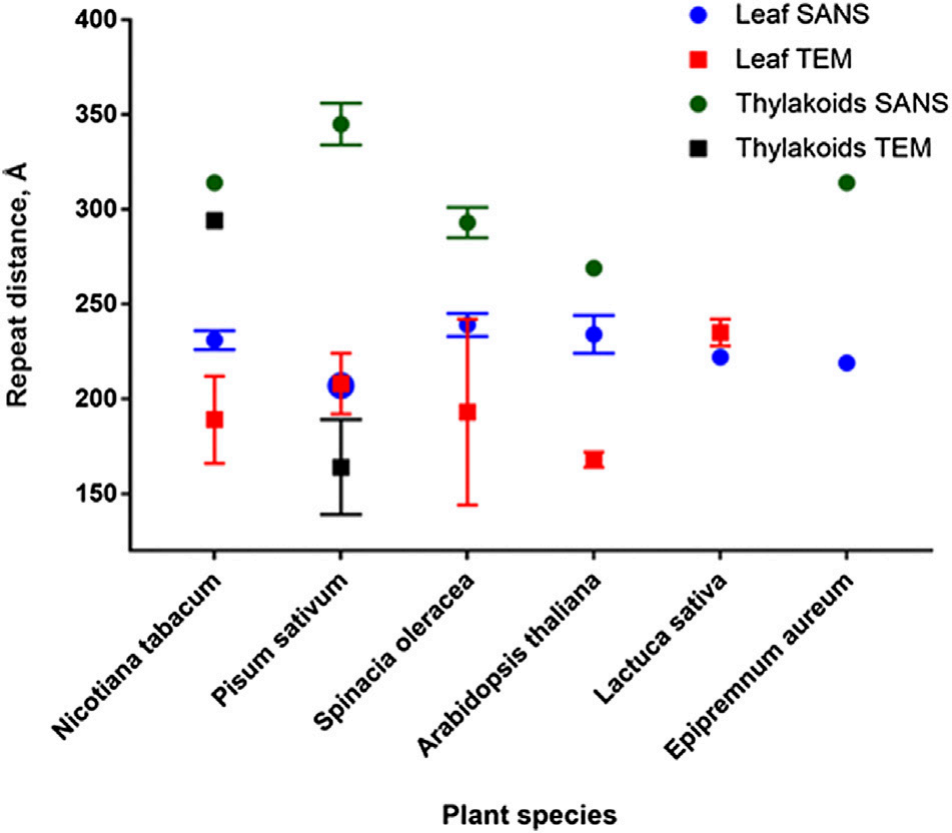
Comparison of repeat distances obtained by SANS and TEM for isolated thylakoids and plant leaves.

As shown in Figure 6, repeat distance values obtained from SANS experiments are generally higher than those obtained from TEM measurements of the same sample (Ünnep et al., 2014b; Eyal et al., 2017). As discussed in Ünnep et al. (2014b), to improve the neutron scattering signal leaves need to be D_2_O infiltrated, which might change the thylakoid organization in the leaf. Slightly expanded thylakoid membranes with D_2_O-infiltrated lumen produce a higher contrast and thus can dominate the scattering, yielding a larger average $D_{R}$ of the scattering peak (Ünnep et al., 2014a). On the other hand, TEM sample preparation involves fixation and dehydration, invoking sample shrinkage. Therefore, $D_{R}$ values *in vivo* can be larger than in TEM micrographs.

## 5 Thylakoid Dynamics

Illumination-induced thylakoid ultrastructure changes are versatile: both shrinkage and expansion of thylakoid repeat distance have been observed in different ionic environments (Deamer and Packer, 1967). Numerous TEM experiments suggest that thylakoid membranes both *in vivo* and *in vitro* contract in response to illumination; this shrinkage is dark-reversible, and its degree depends on light intensity (Murakami and Packer, 1969; Murakami and Packer, 1970a; Murakami and Packer, 1970b; Sundquist and Burris, 1970; Miller and Nobel, 1972; Yamamoto et al., 2013; Yoshioka-Nishimura et al., 2014; Wood et al., 2018). Scattering can also follow thylakoid stacking repeat distance changes—experiments on isolated thylakoids show that thylakoid repeat distance decreases in a matter of minutes when illuminated with white light intensities up to 1,000 μmol photons m^−2^ s^−1^ and is restored when the light is turned off (Nagy et al., 2011; Posselt et al., 2012; Nagy et al., 2013); the higher the applied light intensity, the larger and quicker the $D_{R}$ decrease (Nagy et al., 2013). If illumination intensity on isolated thylakoids is increased to 2,500 μmol photons m^−2^ s^−1^, thylakoid damage occurs and dark readaptation after shrinkage is no longer observed (Posselt et al., 2012). Ultrastructural changes of isolated thylakoids with uncouplers limiting electron transport and pH gradient buildup are not observed upon illumination (Nagy et al., 2013); however, for higher plant thylakoids the role of uncouplers is less clear, and nonphotochemical quenching is proposed to take part in light-induced thylakoid dynamics as well (Ünnep et al., 2020).

Compared to isolated systems, light-induced thylakoid dynamics *in vivo* is more versatile and organism dependent. Several structural outcomes have been observed in plant leaves: thylakoid expansion (Kirchhoff et al., 2011; Yoshioka-Nishimura et al., 2014; Tsabari et al., 2015), thylakoid shrinkage (Miller and Nobel, 1972; Yamamoto et al., 2013; Ünnep et al., 2014b; Tsabari et al., 2015; Wood et al., 2018; Ünnep et al., 2020), and simultaneous thylakoid shrinkage and expansion (Puthiyaveetil et al., 2014; Yoshioka-Nishimura et al., 2014). Apart from that in plants, thylakoid shrinkage was also observed in algal *Porphyra* and *Ulva* cells *in vivo* (Murakami and Packer, 1970a). Light-induced increase of thylakoid repeat distance was observed in *Chlamydomonas* (Nagy et al., 2014), diatoms (Nagy et al., 2012), and cyanobacterial phycobilisome mutant cells (Liberton et al., 2013a), but no $D_{R}$ change was observed in *Synechocystis* sp. PCC 6803 (WT) cells (Nagy, 2011; Liberton et al., 2013a).

As discussed above, SANS data analysis based on peak position readings cannot infer lumen height or thylakoid membrane thickness or their illuminated-induced changes. Therefore, elucidations of dark–light–dark thylakoid dynamics based on scattering data (Liberton et al., 2013a; Ünnep et al., 2020) are so far incomplete. Suitable modeling which investigates the entire scattering curve behavior is of high importance to investigate lumen changes and thylakoid dynamics in general. Furthermore, inelastic neutron scattering can be used to study dynamics of individual membranes. An investigation of dark-adapted and illuminated cyanobacterial thylakoid membrane dynamics shows that the dark-adapted thylakoid membrane is softer before its illumination with 100 µmol photons m^−2^ s^−1^ white light (Stingaciu et al., 2016; Stingaciu et al., 2019). From SANS, thylakoids in *Chlamydomonas* cells are suggested to exhibit the same behavior (Nagy et al., 2014). However, an experiment with a fluorescence probe suggests that spinach thylakoid membrane fluidity is lower in dark-adapted than in low-light-illuminated state (Yamamoto et al., 2013), which calls for a more detailed investigation.

To conclude, the observation of thylakoid (lumen) shrinkage or expansion is only a single facet of thylakoid dynamics *in vivo* and shall not be the sole experimental purpose, as it depends and is probably governed by numerous environmental factors. It has been extensively demonstrated that thylakoid ultrastructure and degree of thylakoid dynamics *in vivo* depend on the organism (Nagy, 2011; Liberton et al., 2013a; Ünnep et al., 2014a; Demmig-Adams et al., 2015; Ünnep et al., 2020), arrangement and composition of photosynthetic proteins and lipids in the thylakoid membrane (Demmig-Adams et al., 2015; Mazur et al., 2019), “previous-growth-history” of a plant (Demmig-Adams et al., 2015; Schumann et al., 2017), illumination spectral quality (Mustárdy et al., 1976; Clausen et al., 2014; Nagy et al., 2014; Bína et al., 2016; Li et al., 2016; Schumann et al., 2017; Ünnep et al., 2020), and illumination intensity (Posselt et al., 2012; Nagy et al., 2013; Yamamoto et al., 2013; Puthiyaveetil et al., 2014; Wood et al., 2019). Understanding the interplay of these factors will yield a much more comprehensive picture of thylakoid dynamics.

## 6 Other Organisms and Prolamellar Bodies

### 6.1 *Phaeodactylum tricornutum*

Intact marine diatom *Phaeodactylum tricornutum* cells have stacked thylakoid membranes, organized in groups of three (Pyszniak and Gibbs, 1992), although cells cultivated under low intensity red-enhanced illumination were shown to increase the number of homogeneously stacked thylakoids (Bína et al., 2016). In these conditions, large thylakoid membrane areas are occupied exclusively by densely packed elliptical PSI-Lhcr supercomplexes. An inhomogeneous photosystem distribution is proposed for *P. tricornutum* thylakoids, where the outer thylakoid lamellae contain more PSI and ATP synthase complexes, compared to the inner membranes of the stacks (Bína et al., 2016).

Day-light-grown *P. tricornutum* cells exhibit a characteristic scattering profile with two characteristic diffraction peaks positioned at *q* = 0.037 Å^−1^ (170 Å) and 0.065 Å^−1^ (97 Å) and with a weak peak in between at *q* 0.052 Å^−1^ (121 Å) [see Figure 1 in Nagy et al. (2012)]. In Nagy et al. (2012), the latter is tentatively assigned to adjacent membrane pairs while no account of the weak central peak is given. In line with the above statements, we conjecture that a full modeling approach accounting for the distinct triplet organization and the possible asymmetric composition of the membranes will account for the full scattering curve.

Nevertheless, qualitative information can still be extracted. Upon white light illumination (150 or 1,200 μmol photons m^−2^ s^−1^), *q* of the peak decreases, indicating an expansion of the stacking—the higher the light intensity, the higher the expansion. This illumination effect was reversible and could not be inhibited by uncouplers, suggesting that thylakoid dynamics are caused by changes in the electrostatic interactions of local electric fields and/or constitutive redistribution of the ions—and not due to pH changes, as in the case of isolated thylakoids (Zimanyi and Garab, 1989). As discussed, light-induced thylakoid expansion in live cells is similar to the thylakoid membrane behavior in intact *Arabidopsis* leaves, which strongly supports the need to analyze thylakoid membrane behavior in a variety of organisms.

In line with experiments on isolated thylakoid membranes, *q*-values of the peaks increase after addition of 100–600 mM sorbitol whereas peak intensity decreases—indicating thylakoid membrane shrinkage in higher osmolarities. Osmoticum-induced shrinkage is reversible—if sorbitol is removed, scattering signal intensity of the first peak is restored, although the intensity of the second peak remains lower. After dark readaptation *q* of the two peaks even decrease to slightly lower values than of nontreated cells, indicating a slight thylakoid membrane swelling during readaptation (Nagy et al., 2012). Similar thylakoid membrane shrinkage is also observed after heat treatment of *P. tricornutum* cells: the entire SANS profile shifts to higher *q* values and peak intensities are decreased.

### 6.2 *Chlamydomonas reinhardtii*

Single-cell green algae *Chlamydomonas* contain well-defined separate regions of stacked and unstacked thylakoid membranes with distinct protein contents and supramolecular structures. *Chlamydomonas* is an attractive organism to study thylakoid ultrastructure and dynamics *in vivo*, especially because of state transitions. Although *Chlamydomonas* thylakoids are organized less regularly (Engel et al., 2015), distinct regions with predominantly grana-like stacks or stroma lamellae are present—an overall organization similar to higher plants, although with a lower number of lamellae in the stacks. From tomography experiments, *Chlamydomonas* thylakoid stacks are composed of 3–10 thylakoids, which have a lateral repeat distance of 224 ± 13 Å. A single thylakoid membrane thickness is 49 ± 5 Å, thylakoid lumen thickness 90 ± 14 Å, and interthylakoid stromal space 36 ± 5 Å (Engel et al., 2015). From SANS experiments, living *Chlamydomonas* cells exhibit a scattering profile with two characteristic diffraction peaks corresponding to *q* 0.033–0.0035 Å^−1^ (180–190 Å) and 0.055 Å^−1^ (114 Å). The first peak/feature is proposed to originate from the repeat distance of stacked thylakoid membranes and the second from the membrane pairs (Nagy et al., 2014). If so, the repeat distance obtained from SANS correlates well with electron tomography data, although the overall appearance of the scattering curve is currently not accounted for and needs to be investigated in more detail to clarify if the offset of the peaks from a lamellar pattern is not simply an effect of the form factor and the low number of layers.

### 6.3 Prolamellar Bodies

We will finish this excursion of scattering work on photosynthetic membranes by returning to the plant prolamellar bodies (PLBs) illustrated in Figure 1A. In contrast to all the other membrane systems described so far, prolamellar bodies are not flat sheet stacks so the modeling approach from Jakubauskas et al. (2019)) is not relevant directly. Instead, the analysis of the scattering requires one to index peaks based on symmetry considerations akin to the analysis routinely performed in, for example, lyotropic liquid crystalline systems. Lachmann and Kesselmeier (1989) classified the isolated PLB ultrastructures according to their internal structure as paracrystalline, spongy, or tubular. Paracrystalline PLBs are well organized and ordered; spongy PLBs maintain elements of the original lattice, but their long-range order is lost; tubular PLBs have tubules from the original structure, but PLBs look torn apart, with no apparent order.

**FIGURE 7:**
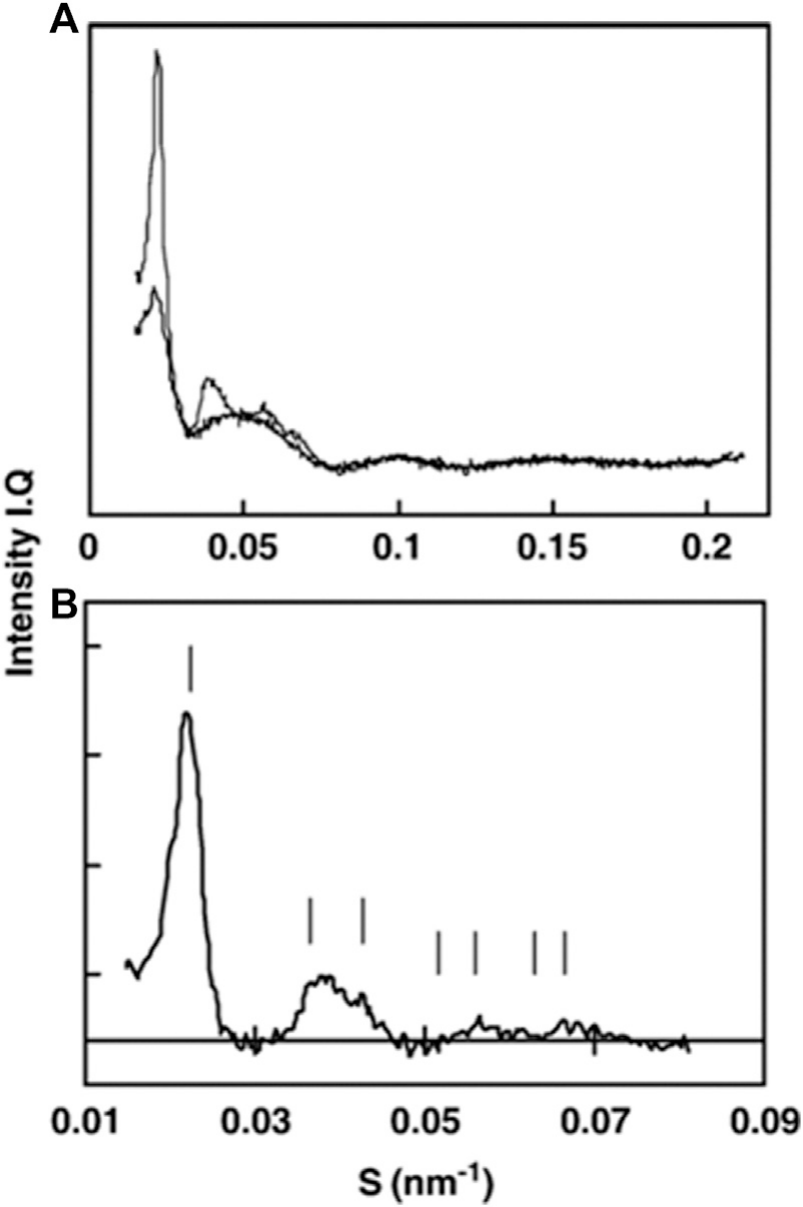
SAXS pattern changes after single freeze–thaw cycle of prolamellar bodies. **(A)** Prior to freezing (thin line) and immediately after melting (thick line). **(B)** Difference between curves from **(A)** indexed to a F*d*3*m* lattice [figure from Selstam et al. (2007)].

X-ray scattering on isolated PLBs was successfully employed by P. Williams and E. Selstam, when they isolated PLBs and used them for parallel SAXS and TEM studies (Williams et al., 1998; Selstam et al., 2007). Judged from TEM pictures, 70–80% of isolated PLBs in the samples were paracrystalline, the remaining PLBs were spongy, with a very low numbers of tubular PLBs or membrane debris from damaged PLBs, although the latter did not interfere with X-ray scattering from highly ordered PLB structures. Concentrated PLB pellets gave rise to X-ray diffraction patterns resembling a F*d*3*m* lattice with unit cell length *a* = 78 nm (see Figure 7). Scattering was then used to study PLB ultrastructure changes with focus on the impact of salts, cryoprotectant, pH, and freeze–thaw cycles (Williams et al., 1998; Selstam et al., 2007). Although promising in essence, PLB studies proved to be technically difficult—mainly, a robust etiolated plant growth setup and sample preparation are necessary to obtain homogeneous PLB preparations for the scattering measurements. Furthermore, to investigate PLB ultrastructure, the sample enclosure itself needs to be light impermeable and the sample needs to be loaded into it under a very low intensity green light, as 1 ms flash of white light can be sufficient to destroy the paracrystalline order of the PLB. One of the possible ways to tackle the issue of PLB sample resolution is the usage of neutron scattering. Here, scattering from lipids is enhanced if isolated PLBs are resuspended in D_2_O-based medium. Furthermore, as is the case for thylakoids, scattering of fresh etiolated and D_2_O-infiltrated leaf stack can be investigated. Since the neutron beam is in most cases larger than that with X-rays, lower concentration of PLBs in the leaf can be compensated by the higher screened sample area and leaf stacking, putatively leading to a comparable signal intensity as for concentrated PLBs investigated by a small X-ray beam. Such an experiment does not require any special sample preparation—PLBs can in principle be investigated in *in vivo* conditions, directly in ethiolated leaves, thus yielding more precise average unit cell values and space group assignment. Ultimately, PLB ultrastructures from various plants and photosynthetic mutants can be investigated and compared, as in Bykowski et al. (2020), and a continuous light-induced PLB disassembly can be ideally followed as well.

## 7 Outlook

Here we have presented an overview of scattering results obtained so far on photosynthetic membranes and advocate for a holistic modeling approach to scattering data as well as the joint utilization of the complementary methods of scattering and microscopy/tomography to study biological samples as close to their native state as possible. Ideally, a study of high biological relevance could investigate thylakoid dynamics in plants with different degrees of thylakoid stacking and diverse photosynthetic and environmental phenotypes: photosynthetic, pigment, or antennae-deficient mutants, draught, and cold-resistant species. Systematically varying white light intensities and using certain wavelengths, causing state transitions in illumination studies, would enable studying the photosynthetic response and changes in thylakoid ultrastructure and evaluating small-scale dynamics of thylakoid membranes, which would greatly benefit biological investigations in plants and cyanobacteria. Furthermore, the entire PLB-to-thylakoid membrane transition could in principle be followed with scattering experiments, which is one of the most spectacular membrane remodelings known in biology.

To conclude, we believe scattering techniques will ultimately enable one to investigate, follow, and model ultrastructural changes of complex biological membrane systems in their native environment in near-second range. In complement to “static” microscopy techniques and together with the fact that comprehensive mathematical models explaining scattering data from complex systems are underway, an advent of new discoveries using scattering methods on complex biological system dynamics is anticipated.
